# Estrogen-mediated inactivation of FOXO3a by the G protein-coupled estrogen receptor GPER

**DOI:** 10.1186/s12885-015-1699-6

**Published:** 2015-10-15

**Authors:** Erin Zekas, Eric R. Prossnitz

**Affiliations:** Department of Internal Medicine and UNM Cancer Center, University of New Mexico Health Sciences Center, Albuquerque, NM 87131 USA

## Abstract

**Background:**

Estrogen (17β-estradiol) promotes the survival and proliferation of breast cancer cells and its receptors represent important therapeutic targets. The cellular actions of estrogen are mediated by the nuclear estrogen receptors ERα and ERβ as well as the 7-transmembrane spanning G protein-coupled estrogen receptor (GPER). We previously reported that estrogen activates the phosphoinositide 3-kinase (PI3Kinase) pathway *via* GPER, resulting in phosphatidylinositol (3,4,5)-trisphosphate (PIP3) production within the nucleus of breast cancer cells; however, the mechanisms and consequences of this activity remained unclear.

**Methods:**

MCF7 breast cancer cells were transfected with GFP-fused Forkhead box O3 (FOXO3) as a reporter to assess localization in response to estrogen stimulation. Inhibitors of PI3Kinases and EGFR were employed to determine the mechanisms of estrogen-mediated FOXO3a inactivation. Receptor knockdown with siRNA and the selective GPER agonist G-1 elucidated the estrogen receptor(s) responsible for estrogen-mediated FOXO3a inactivation. The effects of selective estrogen receptor modulators and downregulators (SERMs and SERDs) on FOXO3a in MCF7 cells were also determined. Cell survival (inhibition of apoptosis) was assessed by caspase activation.

**Results:**

In the estrogen-responsive breast cancer cell line MCF7, FOXO3a inactivation occurs on a rapid time scale as a result of GPER, but not ERα, stimulation by estrogen, established by the GPER-selective agonist G-1 and knockdown of GPER and ERα. GPER-mediated inactivation of FOXO3a is effected by the p110α catalytic subunit of PI3Kinase as a result of transactivation of the EGFR. The SERMs tamoxifen and raloxifene, as well as the SERD ICI182,780, were active in mediating FOXO3a inactivation in a GPER-dependent manner. Additionally, estrogen-and G-1-mediated stimulation of MCF7 cells results in a decrease in caspase activation under proapoptotic conditions.

**Conclusions:**

Our results suggest that non-genomic signaling by GPER contributes, at least in part, to the survival of breast cancer cells, particularly in the presence of ER-targeted therapies involving SERMs and SERDs. Our results further suggest that GPER expression and FOXO3a localization could be utilized as prognostic markers in breast cancer therapy and that GPER antagonists could promote apoptosis in GPER-positive breast cancers, particularly in combination with chemotherapeutic and ER-targeted drugs, by antagonizing estrogen-mediated FOXO3a inactivation.

## Background

Estrogen is the predominant female sex hormone and is involved in an array of physiological processes in addition to reproduction and development of secondary sex characteristics [1], including cardiovascular, immune, endocrine/metabolic and nervous system functions, in both women and men [2]. The most biologically active form of estrogen, 17β-estradiol, is produced primarily in the ovaries of premenopausal females and the testes of males, but secondary sources, such as adipose in postmenopausal women [3], represent alternative sources of estrogen. In females, estrogen regulates mammary growth and development at puberty, throughout the menstrual cycle and during pregnancy and lactation. In fact, breast development in humans represents the only tissue that undergoes the majority of its maturation postnatally, with recurrent expansion and regression/involution throughout life as a result of pregnancy [4, 5]. As a consequence, cell proliferation and apoptosis are under exquisite control, with much of the proliferative response regulated by steroid hormones. Thus, when normal mammary growth regulatory pathways become dysregulated, uncontrolled cell proliferation and loss of apoptosis can lead to breast cancer [4, 6].

Estrogen’s actions, particularly with respect to transcriptional regulation, are mediated in large part by the classical nuclear receptors ERα and ERβ [7]. However, estrogen also mediates rapid cellular signaling events, such as kinase activation (e.g. ERK1/2, Akt), nitric oxide production and calcium mobilization [8]. Although many of these pathways appear to be activated by ERα [9], recent evidence reveals that that G protein-coupled estrogen receptor GPER (previously termed GPR30) also mediates a multitude of rapid signaling events in response to estrogen [10–17] and is important in breast carcinogenesis and metastasis [18, 19] as well as in immune [20, 21], cardiovascular [10, 22, 23], and metabolic/endocrine functions [24–26]. GPER was first demonstrated to be responsible for estrogen’s activation of the MAP kinases ERK1/2 in ERα-and ERβ-negative breast cancer cells, through a mechanism involving the transactivation of epidermal growth factor receptor (EGFR) by metalloproteinase-released HB-EGF [27]. Subsequently, estrogen and tamoxifen were demonstrated to activate PI3Kinase in breast cancer cells and receptor-transfected COS-7 cells *via* GPER, also as a consequence of EGFR transactivation [28]. Interestingly, ERα was also capable of mediating PI3Kinase activation in ERα-transfected COS cells but only in response to estrogen and not tamoxifen stimulation, and *via* a pathway that did not involve EGFR transactivation [28]. Finally, although the direct activation of EGFR with EGF led to the activation of PI3Kinase with resulting PIP3 production at the plasma membrane, as indicated by the plasma membrane localization of the PIP3 reporter Akt-PH-RFP (the PIP3-binding PH domain of Akt fused to RFP), activation of either ERα with estrogen or GPER with estrogen or tamoxifen, led to the nuclear accumulation of Akt-PH-RFP, suggesting that PIP3 production was occuring in the nucleus and might lead to the activation of a nuclear pool of Akt that in turn would mediate responses distinct from the plasma membrane pool of Akt [28].

The enzyme PI3Kinase converts the membrane phospholipid phosphatidylinositol-4,5-bisphosphate (PIP2) into phosphatidylinositol-(3,4,5)-trisphosphate (PIP3). PI3Kinase consists of a catalytic domain and a regulatory domain. The two ubiquitously expressed catalytic domains, p110α and p110β, are usually coupled to their respective regulatory subunits p85α and p85β [29]. The p110α subunit has been demonstrated to have a role in growth factor and metabolic signaling as well as being selectively mutated and overexpressed in a variety of cancers [30]. The p110β subunit, however has been reported to be expressed in the nucleus and to be involved in DNA replication, S phase progression, and DNA repair [31–33].

Following PIP3 production by PI3Kinase activation, PIP3 recruits Akt and PDK to membranes, leading to Akt phosphorylation and activation by PDK. Activated Akt has many substrates including members of the forkhead box O (FOXO) class of transcription factors, which are involved in cell fate decisions, proliferation, and metabolism [34, 35]. Because their functions are regulated by pathways found to be dysregulated in cancer, certain FOXO proteins have been described as tumor suppressors [36–40]. The major mechanism through which FOXO transcription factor activity is regulated in response to external stimuli is *via* changes in subcellular localization. The FOXO family member FOXO3a specifically promotes the transcription of proapoptotic genes, such as Bim, p21 and p27, and is inactivated through phosphorylation by Akt, among many other kinases [35, 41]. This leads to its translocation from the nucleus to the cytoplasm, followed by ubiquitination and proteasome-mediated degradation, resulting in a decrease in the expression of proapoptotic genes [34]. FOXO3a localization has been employed to assess the proliferative/prosurvival vs. proapoptotic status of cells [42, 43]. Predominantly nuclear localization of FOXO3a suggests a proapoptotic state, whereas cytosolic localization of FOXO3a suggests an anti-apoptotic state. In breast cancer cells, FOXO3a has been shown to localize to the nucleus in response to chemotherapeutic drugs, such as doxorubicin, under otherwise proliferative conditions [44]. Furthermore, in patient tissue samples, nuclear localization in luminal-like breast cancers has been associated with a good prognosis [45]. However, the direct and rapid effects of estrogen on FOXO3a localization and activity have not been investigated.

In the present study, we utilized the human breast cancer cell line MCF7, which is highly dependent upon estrogen for growth and survival, to test whether signaling by estrogen modulates FOXO3a localization and thus activity. Furthermore, MCF7 cells express both ERα and GPER [46], as well as ERβ [47], providing an ideal environment to determine which estrogen receptor might regulate FOXO3a activity and to examine the mechanisms involved.

## Methods

### Cell culture

The human breast cancer cell line, MCF7 (obtained from ATCC) was maintained in Dulbecco’s modified eagle’s medium (DMEM) (Sigma) with 1 % Penicillin/Streptomycin/Glutamine Solution (100×, Thermo Scientific) and 10 % Fetal Bovine Serum (Thermo Scientific). Where serum starvation is indicated, DMEM was replaced with DMEM/F-12 (50/50) without phenol red (Cellgro, Mediatech) supplemented with 1 % Penicillin/Streptomycin/Glutamine Solution.

### Inhibitors and antibodies

LY294002 (CalBiochem) was used as a broad-spectrum inhibitor of all PI3Kinase isoforms. PIK-75 (Chemdea) was used to inhibit the p110α isoform of the PI3Kinase catalytic subunit, while TGX-221 (Chemdea) selectively inhibits the p110β isoform. The EGFR inhibitor Tyrphostin AG1478 (Calbiochem) was used to inhibit the EGFR’s tyrosine kinase activation. ERα antibody (Santa Cruz Biotechnology) and a GPER polyclonal antibody raised against a peptide from the human GPER C-terminus as previously described [28] were used to assess receptor expression. Actin antibodies were from Thermo Scientific.

### Transfections and translocation assays

**Fig. 1:**
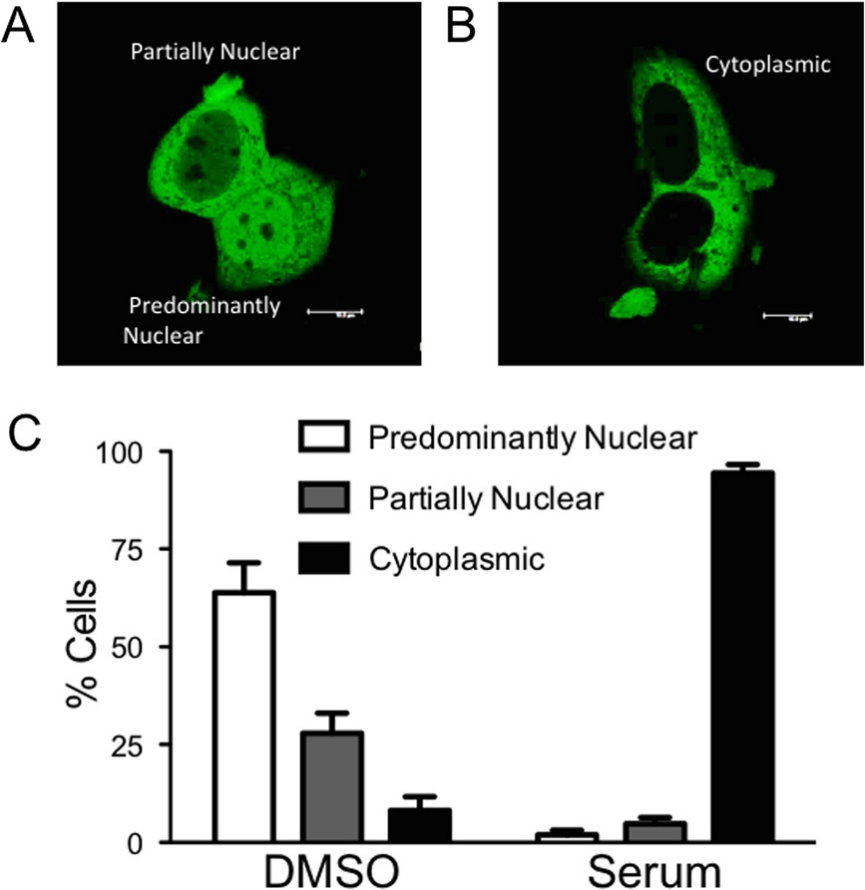
FOXO3-GFP translocates to the cytomplasm upon serum treatment. **a** Representative image of predominantly nuclear and partially nuclear localization of FOXO3-GFP in MCF7 cells transfected with FOXO3-GFP and cultured in serum-free medium for 24 h. **b** Representative image of cytoplasmic localization of FOXO3-GFP in MCF7 cells, prepared as in (**a**), but treated with serum for 15 min. **c** Quantitation of FOXO distribution. Serum-starved, FOXO3-GFP-transfected MCF7 cells were treated with either DMSO (0.1 %) or serum (1:1000 with 0.1 % DMSO) for 15 min. Based on the classification scheme defined in (**a**) and (**b**) and in Results, cells were assigned to the indicated categories and the percentage of cells in each category determined. Results are reported as mean +/− s.e.m. from at least 3 experiments

The FOXO3-GFP plasmid was a generous gift from Dr. Marten P. Smidt (University of Amsterdam) and was generated as described [42]. MCF7 cells were seeded at ~20,000 K cells per well on 12 mm coverslips in a 24 well plate 24 h prior to transfection. Cells were transfected with 0.6 μg of FOXO3-GFP using the Lipofectamine 2000 Transfection reagent (Invitrogen) following the manufacturer’s protocol. Approximately 24 h after transfection cells were serum starved for 24 h followed by stimulation with ligands/inhibitors as indicated. The cells were then fixed with 2 % PFA in PBS, washed, mounted in Vectashield and analysed by confocal imaging on a Leica SP5 microscope. Approximately 50 transfected cells per treated coverslip were analyzed for subcellular localization of FOXO3-GFP. Localization was defined as either: predominantly nuclear, partially nuclear, or cytoplasmic (see Fig. 1).

### siRNA-mediated knockdown

For selective knockdown of either ERα or GPER, MCF7 cells were seeded at ~20,000 K cells per well on 12 mm coverslips in a 24 well plate 24 h prior to transfection. Small-interfering RNA (siRNA) obtained from Dharmacon RNAi Technologies included: siGPER (ONTARGET plus SMARTpool siRNA (L-005563-00)), siERα (ONTARGET plus SMARTpool siRNA (L-003401-00) Human ESR1), and siControl (ON-TARGETplus siControl Non-Targeting siRNA (D-001810-02)). Each was transfected (50 pmol per well) using Lipofectamine 2000 and 24 h after siRNA transfection, 0.6 μg of FOXO3-GFP plasmid was transfected into each well as above. The following day, cells were serum starved for 24 h and treated as specified. The cells were fixed, visualized and analyzed as above.

### Caspase activation

Capsase 7 activation was assessed using the Magic Red® Caspase 3/7 Detection Kit (Immunochemistry Technologies). MCF7 cells were seeded onto 12 mm coverslips in a 24 well plate. Approximately 24 h after seeding, treatments were initiated for 2 or 3 days employing serum-free/phenol red-free medium. At the end of each time point, cells were incubated with the Magic Red substrate solution diluted as described by manufacturer’s protocol for 1 h at 37 °C, washed twice with PBS and fixed with 4 % PFA. Cells were washed twice and stained with TO-PRO-3 (Life Technologies) for 10 min at room temperature in the dark and then washed twice with PBS. The coverslips were mounted in Vectashield and analyzed by confocal imaging on a Leica SP5 microscope. At least three images per treatment per experiment were analyzed utilizing Slidebook (Intelligent Imaging Innovations) as follows. The total (sum) fluorescence intensity of the Cy3 (Magic Red) channel for each image was recorded and divided by the number of cells per image (assessed by TO-PRO-3). Each averaged treatment was normalized to its corresponding averaged negative control (DMSO) for that experiment.

### Western blotting

MCF7 cells were seeded to 60-80 % confluency in 60 mm dishes and serum starved 24 h prior to the indicated treatments. Following treatment, cells were washed twice with cold PBS and scraped into lysis buffer: RIPA buffer containing NP-40 supplemented with sodium fluoride (50 mM), sodium orthovanadate (1 mM), phenylmethylsulfonylfluoride (1 mM), 0.1 % SDS, 0.5 % sodium deoxycholate and protease cocktail (1×). Cell lysate protein concentration was determined by Bradford protein assay (Bio-Rad). Equal protein concentrations per lysate were loaded on a 4-20 % Precise Tris-Glycine Gels (Thermo Scientific) and transferred to polyvinylidene difluoride membranes (Millipore). Membranes were blocked in 5 % Blotting Grade Blocker Non-Fat Dry Milk (Bio-Rad) for 1 h at room temperature and then incubated with primary antibodies (1:500 for ERα; 1:3000 for GPER; 1:10,000 for actin) in 3 % BSA overnight at 4 °C. The blots were then incubated with horseradish peroxidase-conjugated goat anti-rabbit IgG (1:3000) or goat anti-mouse IgG for actin (1:5000) in 3 % BSA for 1 h at room temperature and developed using Supersignal West Pico Chemiluminescent Substrate (Thermo Fisher). Films were scanned and quantified using ImageJ software (National Institutes of Health).

### Statistical analysis

Statistical analysis was performed using GraphPad Prism version 5 with a one-way analysis of variance (ANOVA). Subsequent pairwise comparisons between different treatment groups were determined using Dunnett’s or Newman Keul’s post-hoc analysis. Data represents the mean ± SEM of three or more separate experiments. *P*-values less than 0.05 were considered to be significant. In order to determine significance in siRNA experiments between siControl and siGPER or siERα, a two-way ANOVA analysis was performed utilizing a Bonferroni post-hoc test.

## Results

### Estrogen stimulation leads to rapid FOXO3a translocation to the cytoplasm

Despite being thought of as a primarily cytoplasmic kinase functioning at the plasma membrane, Akt has many known targets within the nucleus [48]. In particular, Akt has been demonstrated to phosphorylate the proapoptotic transcription factor, FOXO3a. When FOXO3a is active (i.e. unphosphorylated), it resides predominantly within the nucleus functioning as a transcription factor stimulating the expression of proapoptotic genes [35, 36]. In the presence of growth factor signaling that activates the PI3Kinase pathway, FOXO3a is phosphorylated by Akt and translocates from the nucleus to the cytoplasm, where it is subsequently degraded [35, 36]. In order to determine whether estrogen-mediated activation of the PI3Kinase pathway regulates FOXO3a localization, we employed a FOXO3-GFP fusion protein [42]. FOXO3, the murine ortholog of human FOXO3a, shares high sequence homology as well as the same Akt phosphorylation sites and regulatory properties with its human ortholog [42].

To examine the ability of FOXO3-GFP to translocate upon cell stimulation, we transfected MCF7 cells with FOXO3-GFP and monitored its localization after 24 h of serum starvation followed by brief (15 min) stimulation with serum (Fig. 1). In general, we observed FOXO3-GFP localization in three distinct patterns: predominantly nuclear, partially nuclear, and cytoplasmic (Fig. 1a and b). Predominantly nuclear (Fig. 1a, cell to lower right) refers to cells where nuclear FOXO3-GFP intensity is clearly stronger than the cytoplasmic intensity, while partially nuclear (Fig. 1a cell on left) refers to cells with greater cytoplasmic localization, but with clearly observable FOXO3-GFP in the nucleus. Cells classified as cytoplasmic exhibit little to no detactable FOXO3-GFP in the nucleus (Fig. 1b). The percentage of each localization pattern in the population of transfected cells was assessed as an indicator of FOXO3 activation status (Fig. 1c). The majority of control (serum starved) DMSO-treated cells exhibited predominantly nuclear localization (predominantly or partially nuclear) with only about 10 % of cells exhibiting strongly cytoplasmic localization, suggesting that FOXO3 is active under these conditions. Upon stimulation with serum, approximately 95 % of cells exhibited a strongly cytoplasmic localization of FOXO3-GFP, demonstrating translocation to the cytoplasm and suggesting FOXO3 has become inactivated, presumably through phosphorylation by kinases such as Akt.

**Fig. 2:**
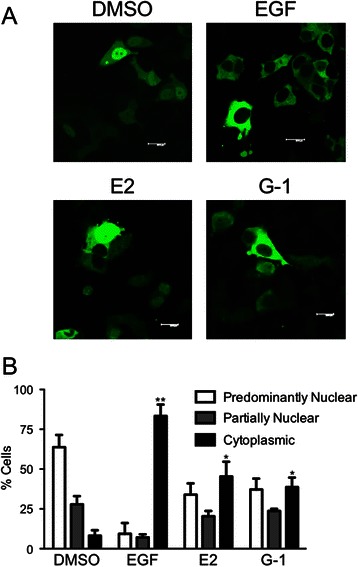
Estrogen activation leads to FOXO3a translocation. **a** Representative images of MCF7 cells transfected with FOXO3-GFP, starved of serum for 24 h and treated with 0.1 % DMSO, 50 ng/ml EGF, 50 nM estrogen, or 100 nM G-1 for 15 min. **b** Quantitation of the localization pattern of FOXO3-GFP-expressing cells from (**a**) as defined in Fig. 1. Results are reported as mean +/− s.e.m. from at least 3 experiments. *, *p* < 0.05; **, *p* < 0.01 vs. cytoplasmic localization of the DMSO control

In order to determine the effects of estrogen stimulation on FOXO3-GFP inactivation, FOXO3-GFP-transfected MCF7 cells were treated with non-selective agonist 17β-estradiol (estrogen) or the GPER-selective agonist G-1 [16] and FOXO3-GFP localization patterns assessed (Fig. 2). Furthermore, instead of using serum as above, which contains a complex array of growth factors and cell stimulants, we tested whether EGF alone was capable of recapitulating the effects observed with serum. As EGFR activation typically leads to PI3Kinase activation, we speculated that EGF stimulation would lead to FOXO3a inactivation [49]. EGF induced an almost quantitative translocation of FOXO3-GFP from a predominantly and partially nuclear localization to a cytoplasmic localization (Fig. 2a and b). Estrogen-and G-1-treated cells exhibited a decrease in the percentage of cells with a predominantly nuclear localization of FOXO3-GFP with a concomitant increase in the percentage of cells displaying a cytoplasmic localization, although not to the same extent as EGF (or serum).

**Fig. 3:**
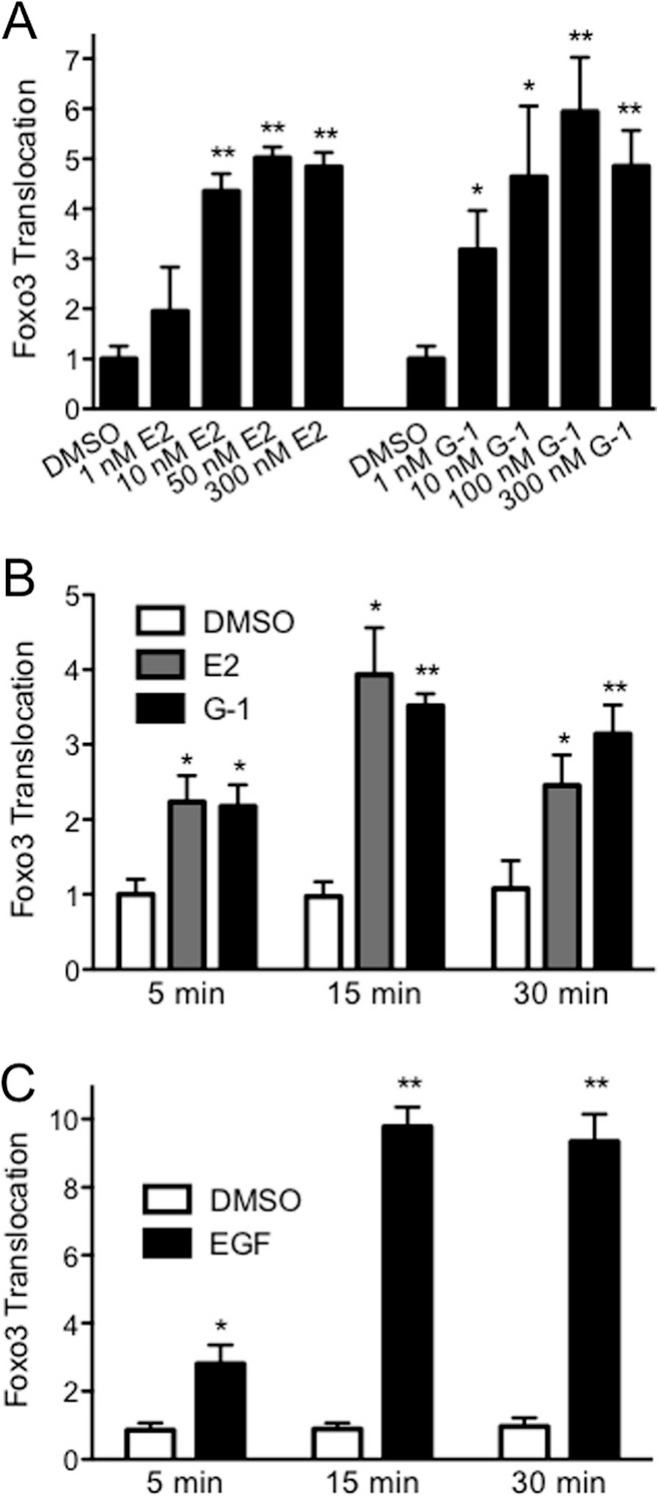
Dose and temporal responses of FOXO3-GFP translocation. **a** MCF7 cells transfected with FOXO3-GFP were serum starved for 24 h and treated with the indicated concentrations of estrogen or G-1 for 15 min. **b** and **c** MCF7 cells transfected with FOXO3-GFP, serum starved for 24 h and treated with 0.1 % DMSO, 50 nM estrogen, 100 nM G-1 (**b**) or 50 ng/ml EGF (**c**) for 5, 15 or 30 min. Results are reported as mean +/− s.e.m. from at least 3 experiments. *, *p* < 0.05; **, *p* < 0.01 vs. DMSO control

In order to simplify the presentation of FOXO3-GFP translocation in the remaining experiments, the cytoplasmic localization for any given treatment is presented as the fold increase compared to the DMSO vehicle control, as this accurately reflects the changes in the overall localization state of FOXO3. In order to determine the dose dependence of FOXO3-GFP inactivation on estrogen and G-1 concentration, dose response profiles for estrogen and G-1 were performed (Fig. 3a). Stimulation by concentrations of G-1 and estrogen as low as 1 and 10 nM, respectively, yielded significant increases in FOXO3-GFP translocation with maximal responses for G-1 and estrogen obtained at ~100 nM and 50 nM, respectively, which are the concentrations utilized throughout the remainder of this study. Since rapid signaling events typically occur within approximately 30 min, FOXO3-GFP inactivation by estrogen and G-1 was monitored over a 30-minute period. Whereas FOXO3-GFP translocation could be observed as early as 5 min following stimulation, maximal responses to both ligands were observed by 15 min (Fig. 3b). These results are consistent with the translocation of FOXO3-GFP induced by EGF, which was also detected as early as 5 min following stimulation, with maximal stimulation observed by 15 min (Fig. 3c). These results thus represent the first report of rapid inactivation of FOXO3 in response to estrogen stimulation in breast cancer cells.

### Estrogen-stimulated translocation of FOXO3-GFP is mediated by GPER

**Fig. 4:**
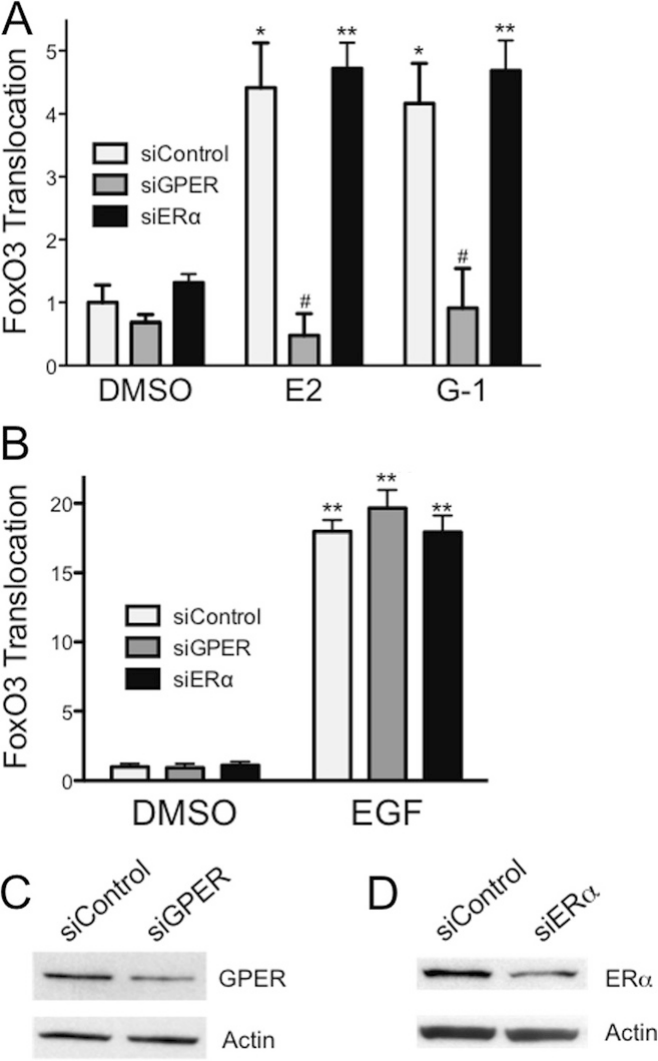
GPER, but not ERα, is required for estrogen-and G-1-mediated translocation of FOXO3-GFP. MCF7 cells were transfected with siRNA targeting either GPER (siGPER) or ERα (siERα) or a non-targeting siRNA (siControl), followed by transfection with FOXO3-GFP. Transfected cells were serum starved for 24 h prior to treatments. **a** Cells were treated with 0.1 % DMSO, 50 nM estrogen or 100 nM G-1 for 15 min. **b** Cells were treated with 0.1 % DMSO or 50 ng/ml EGF for 15 min. **c** and **d** Representative Western blots of cell lysates collected at the same time as the treatments in (**a**) and (**b**). Results are reported as mean +/− s.e.m. from at least 3 experiments. *, *p* < 0.05; **, *p* < 0.01 vs. DMSO control of the matched siRNA. #, *p* < 0.05 vs. the ligand-matched siControl treatment

MCF7 cells express three estrogen receptors (GPER, ERα and ERβ), all of which are capable of binding estrogen [28, 50]. Because FOXO3-GFP undergoes cytoplasmic translocation in response to estrogen and G-1 treatment, we next sought to determine which estrogen receptor was responsible for estrogen-mediated translocation of FOXO3-GFP. MCF7 cells were consecutively transfected with siRNA targeting either GPER or ERα and FOXO3-GFP, and FOXO3-GFP translocation in response to estrogen and G-1 was assessed. Knockdown of GPER significantly reduced the ability of both estrogen and G-1 to stimulate FOXO3-GFP translocation (Fig. 4a), as evidenced by a significant decrease in the percentage of cells with cytoplasmic FOXO3-GFP localization. This suggests, together with the fact that G-1, a selective GPER agonist, also stimulates FOXO3-GFP translocation, that GPER represents the estrogen receptor responsible for estrogen-mediated (as well as G-1-mediated) inactivation of FOXO3-GFP. Importantly, knockdown of GPER did not affect the ability of EGF to initiate FOXO3-GFP translocation, suggesting GPER-mediated signaling is upstream of EGFR-mediated signaling (Fig. 4b). To determine whether ERα might also play a role in the translocation of FOXO3-GFP in response to estrogen, ERα expression was similarly knocked down. Decreasing ERα expression displayed no effect on the ability of estrogen to mediate FOXO3-GFP translocation, suggesting either that it is not involved or that very low expression levels of ERα are sufficient to mediate signaling (Fig. 4a). Simliar to GPER knockdown, EGF-mediated FOXO3-GFP translocation was not affected by ERα knockdown. Knockdown of both GPER and ERα was confirmed by Western blot (Fig. 4c and d). Together, these results reveal that GPER plays an essential role in the rapid translocation of FOXO3 from the nucleus to the cytoplasm upon estrogen stimulation of MCF7 cells.

### ERα antagonists stimulate FOXO3-GFP translocation in MCF7 cells *via* GPER

**Fig. 5:**
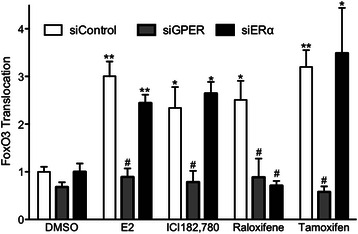
Estrogen receptor antagonist-stimulated FOXO3-GFP translocation in MCF7 cells requires GPER. MCF7 cells were transfected with either siControl, siGPER or siERα and FOXO3-GFP followed by serum starvion for 24 h prior to treatments as in Fig. 4. Cells were treated with 0.1 % DMSO, 50 nM estrogen, or 1 μM ICI182,780, raloxifene or 4-hydroxytamoxifen (Tamoxifen). Results are reported as mean +/− s.e.m. from at least 3 experiments. *, *p* < 0.05; **, *p* < 0.01 vs. DMSO control of the matched siRNA. #, *p* < 0.05 vs. the ligand-matched siControl treatment

GPER has been reported to be involved in drug resistence in response to the SERM tamoxifen [51–54], the ability of tamoxifen to stimulate proliferative signaling and cell migration in endometrial cancer cells [55–57], as well as proliferative signaling and cell adhesion observed in response to the SERD ICI182,780 [55, 58]. Therefore, we sought to determine whether ICI182,780, tamoxifen and an additional SERM, raloxifene could modulate FOXO3-GFP localization in MCF7 cells and if so, which estrogen receptors were involved. ICI182,780, raloxifene and tamoxifen all stimulated FOXO3-GFP translocation to the cytoplasm in a significant percentage of MCF7 cells transfected with control siRNA (Fig. 5). However, knockdown of GPER abrogated FOXO3-GFP translocation by ICI182,780 tamoxifen and raloxifene, consistent with GPER being the estrogen receptor responsible. Knockdown of ERα had no effect on the translocation of FOXO3-GFP by ICI182,780 or tamoxifen, but surprisingly did prevent translocation induced by raloxifene (Fig. 5). These results indicate not only that the extent of ERα knockdown is sufficient to effect a cellular change in responsiveness, but also that whereas GPER appears to be solely responsible for the effects of tamoxifen and ICI182,780, raloxifene-mediated effects require both ERα and GPER.

### FOXO3-GFP translocation requires PI3Kinase activity and EGFR transactivation

**Fig. 6:**
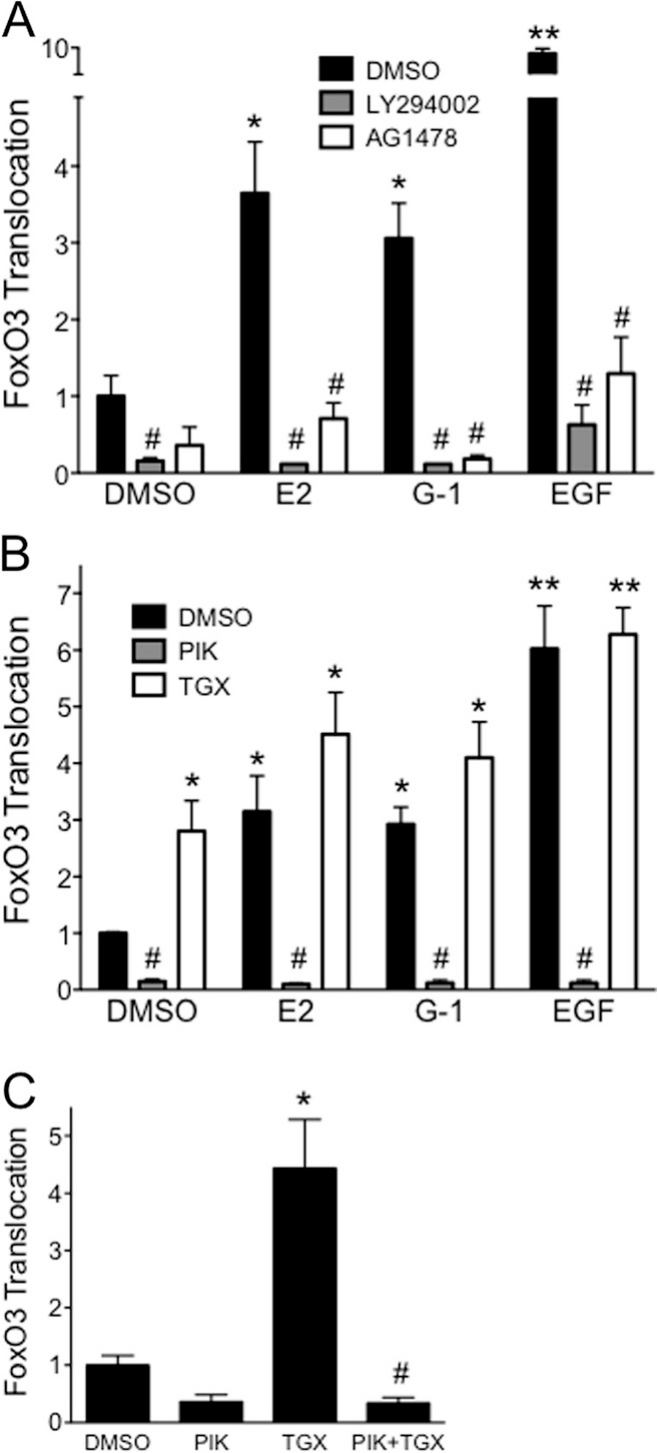
FOXO3-GFP translocation requires the p110α subunit of PI3Kinase and transactivation of the EGFR. **a** FOXO3-GFP-transfected MCF7 cells were serum starved for 24 h prior to the following treatments. Cells were treated with either 50 nM estrogen, 100 nM G-1 or 50 ng/ml EGF for 15 min; where indicated cells were pretreated with 10 μM LY294002 (a broad PI3Kinase inhibitor), 250 nM AG1478 (EGFR inhibitor) or vehicle (DMSO) for 30 min. **b** Cells were treated with estrogen, G-1 or EGF as in (**a**) following pretreatment with 100 nM PIK-75 (a p110α-selective inhibitor), 100 nM TGX-221 (a p110β-selective inhibitor) or vehicle (DMSO) for 30 min as indicated. For (**a**) and (**b**), results are reported as mean +/− s.e.m. from at least 3 experiments. *, *p* < 0.05; **, *p* < 0.01 vs. DMSO control. #, *p* < 0.05 vs. the (ligand-matched) DMSO treatment. **c** MCF7 cells were treated with 100 nM PIK-75, 100 nM TGX-221 or a combination of both inhibitors, in the absence of any ligand, as in (**b**). Results are reported as mean +/− s.e.m. from 3 experiments. *, *p* < 0.05 vs. DMSO control. #, *p* < 0.05 vs. treatment with TGX alone

It has been previously established that GPER stimulation leads to PI3Kinase activation and that EGFR transactivation is required as an intermediate in this signaling pathway [28, 59]. To confirm the role of PI3Kinase in the translocation of FOXO3-GFP in our model, MCF7 cells transfected with FOXO3-GFP were preincubated with the broad spectrum PI3Kinase inhibitor LY294002 (LY) and subsequently treated with estrogen, G-1 and EGF (Fig. 6a). LY294002 abrogated FOXO3-GFP translocation by each ligand, establishing that PI3Kinase is required as a signaling intermediate. Furthermore, the involvement of EGFR was assessed employing the EGFR inhibitor AG1478, which, similar to PI3Kinase inhibition, significantly reduced FOXO3-GFP transloation induced by estrogen, G-1 and EGF (Fig. 6a).

### The p110α subunit of PI3Kinase mediates FOXO3-GFP translocation while p110β inhibition enhances p110α activity

The class I_A_ subset of PI3Kinases consists of a catalytic subunit (p110) and a regulatory subunit (p85). The two ubiquitously expressed PI3Kinase isoforms, p110α and p110β, have multiple yet distinct functions [31]. In order to determine which PI3Kinase isoform is responsible for inducing FOXO3-GFP translocation as a result of GPER stimulation, cells were preincubated with either PIK-75, which selectively inhibits p110α, or the p110β-selective inhibitor, TGX-221 (Fig. 6b). PIK-75 potently inhibited FOXO3-GFP translocation by EGF, estrogen and G-1, suggesting that p110α activity is involved in FOXO3 phosphorylation and inactivation. Surprisingly, TGX-221 enhanced FOXO3-GFP translocation, even when it was added to cells as a control in the absence of a stimulating ligand. We therefore speculated that a balance or cross-interaction may exist between the two p110 isoforms such that inhibiting p110β increases p110α activity. To test this, we incubated MCF7 cells expressing FOXO3-GFP with both TGX-221 and PIK-75, hypothesizing that if TGX-221 inhibition of p110β results in the activation of p110α, then this activity should be inhibited upon inclusion of the p110α-specific inhibitor (Fig. 6c). Indeed, inhibiting both p110 catalytic subunits ablated the tranlsocation of FOXO3-GFP, suggesting that inhibiting p110β results in an upregulation p110α activity. Together, these results reveal that estrogen-mediated stimulation of MCF7 cells results in GPER-mediated transactivation of the EGFR, which in turn activates a PI3Kinase complex containing p110α, resulting in the phosphorylation, translocation and presumably inactivation of FOXO3a.

### Estrogen and G-1 promote survival of MCF7 cells

**Fig. 7:**
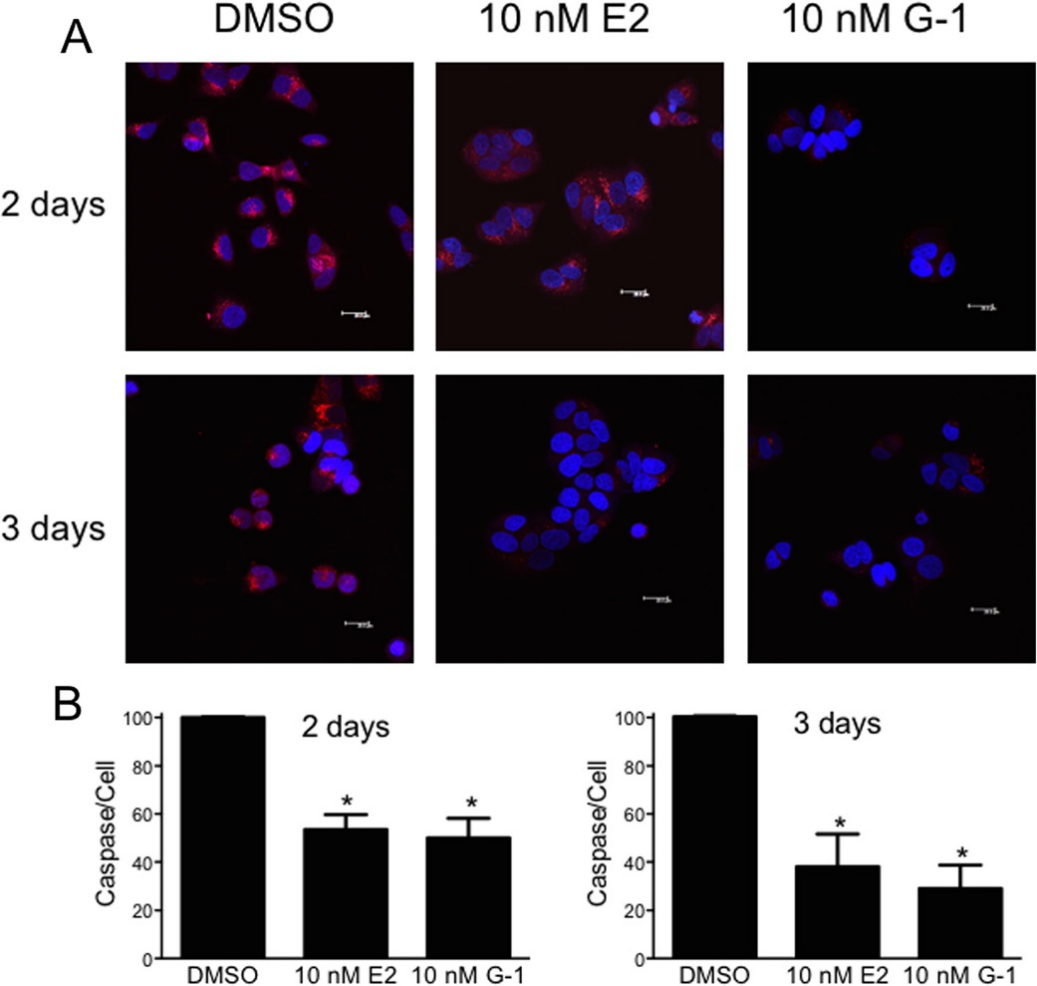
Estrogen and G-1 stimulation of MCF7 cells reduces caspase activation. MCF7 cells, under serum-free conditions, were treated with 10 nM estrogen or G-1 for either 2 or 3 days as indicated. Following treatment, cells were evaluated for caspase 7 activation employing a fluorogenic caspase substrate. **a** Representative images of each treatment at the indicated time point. Accumulation of the fluorescent product (red) is a result of caspase activation; blue, TO-PRO-3 staining of nuclei. **b** Images were analyzed for average fluorescence intensity on a per cell basis, and normalized to the intensity of the DMSO control. *, *p* < 0.05 relative to DMSO control

As estrogen is required for MCF-7 cell proliferation, we hypothesized that estrogen- as well as G-1-mediated phosphorylation, translocation and ultimately inactivation of FOXO3a should shift MCF7 cells towards a more prosurvival state since FOXO3a is generally acknowledged as a proapoptotic transcription factor. To assess shorter-term cell survival, as opposed to long-term proliferation, we measured caspase activation following short-term (2-3 day) serum (and thus estrogen and growth factor) deprivation, as an approach to induce a pro-apoptotic state. Following 2 days of serum starvation, estrogen, as anticipated due to its growth promoting capacity in MCF7 cells, is capable of significantly reducing the level of caspase activation, an effect that is more pronounced following 3 days of starvation (Fig. 7a and b). Importantly, G-1 is also capable of reducing caspase activation to a similar extent as estrogen, suggesting that it is the rapid signaling aspects of estrogen stimulation that likely oppose the pro-apoptotic effects of estrogen/growth factor withdrawal. Although the assay we employed is capable of detecting activation of both caspases 3 and 7, since MCF7 cells are deficient in caspase 3 [60], the detected activity must be due to caspase 7 activation. These results demonstrate that, as anticipated, exposure to estrogen reduces caspase activation in MCF7 breast cancer cells under serum-deprived conditions. However, because G-1 similarly reduces caspase activation, we conclude that rapid signaling by GPER likely plays an important role in the short-term survival effects initiated by estrogen and resulting in FOXO3a phosphorylation and inactivation.

## Discussion

FOXO3a is a critical regulator of cell survival and proliferation; however, its rapid regulation in breast cancer cells, particularly by estrogen, has not been reported. Several reports have demonstrated in MCF7 cells, the ability of chemotherapeutic drugs to induce FOXO3a activation (i.e. accumulation of FOXO3a in the nucleus) [44, 61]. FOXO3a activation results in an increase in proapoptotic protein expression and provides a mechanism for how these drugs promote apoptosis in breast and other cancer cells [37, 39, 40]. The steroid hormone estrogen facilitates progression of hormone-sensitive tumors, including breast cancer, through its classical nuclear receptors by transcriptional regulation resulting in cell survival and proliferation [62]. However, estrogen also mediates rapid signaling and growth effects through an additional estrogen receptor, GPER [10, 19]. These signaling events include the activation of MAPKs [27] as well as activation of the PI3Kinase pathway [28, 59]. The specific downstream effects of PI3Kinase activation by estrogen have not been extensively studied. However, it has been established that PI3Kinase activation by growth factor receptors in general can stimulate Akt to phosphorylate and inactivate FOXO3a, thereby excluding it from the nucleus, leading to its degradation, and the downregulation of proapoptotic gene expression [34]. Here we provide a mechanism by which the activation of GPER by estrogen, as well as the GPER-selective agonist G-1, can lead to PI3Kinase/Akt activation and subsequently the inactivation of FOXO3a, resulting in enhanced survival.

We have previously established that activation of GPER by estrogen, tamoxifen and G-1, as well as estrogen-mediated activation of ERα, can lead to the accumulation of PIP3 in the nucleus, with the nuclear accumulation of PIP3 requiring activation of both PI3Kinase and the EGFR [15, 16, 28]. These experiments were performed in COS-7 cells transiently expressing either ERα or GPER [28] as well as SKBR3 breast cancer cells [28] and Hec50 endometrial cancer cells [55], both of which endogenously express only GPER. In order to investigate this pathway utilizing a more physiologically relevant model of ERα-positive breast cancer, we employed MCF7 cells, which express both ERα and GPER. FOXO3a overexpression has been reported to suppress estrogen-dependent cell proliferation and tumor growth in MCF7 cells [63]. A recent report has further identified ERα as a key regulator of FOXO3a in MCF7 cell motility and invasiveness through the modulation of caveolin expression [64]. In this latter study, overexpression of FOXO3a led to an inhibition of cell migration, invasion and anchorage-independent colony formation. In both the absence and presence of FOXO3a overexpression, estrogen stimulation (further) inhibited migration and invasion, while enhancing colony formation, as previously reported [65]. This result is not unexpected given the reciprocal regulation of migration and proliferation [66]. Interestingly, although knockdown of ERα with siRNA reduced absolute colony formation in both the absence and presence of estrogen, there remained a potent induction of colony formation by E2 in ERα-depleted cells, suggesting the actions of another estrogen receptor. However, upon ERα depletion, FOXO3a overexpression led to an increase in colony formation, the opposite of that observed in ERα-replete cells. Furthermore, in contrast to ERα-positive MCF7 cells, overexpression of FOXO3a in ERα-negative MDA-MB-231 cells led to enhanced migration, invasion and colony formation, the latter in contradistinction to the expected proapoptotic role of FOXO3a. Together, these results suggest a complex interplay between ERα and FOXO3a, further complicated by ERβ having also been reported to interact with FOXO3a in MCF7 cells [63], on the estrogen-mediated regulation of cellular functions including migration, invasion and growth.

Because the above results suggest that the net effects of FOXO3a are highly dependent on the levels of FOXO3a present in the nucleus, we sought to explore the effects of estrogen stimulation on FOXO3a trafficking and localization. Consistent with our previous observations of PI3Kinase activation and PIP3 accumulation in the nucleus, we observed rapid translocation of FOXO3 from the nucleus to the cytoplasm upon estrogen stimulation, a process that was dependent only upon GPER expression and not ERα expression. Further supporting the role of GPER in mediating this estrogen-stimulated effect, the GPER-selective ligand was equally efficacious to estrogen in mediating the rapid translocation. This pathway involved the transactivation of the EGFR, a mechanism now well associated with GPER signaling, and PI3Kinase (specifically p110α) signaling. As the majority of GPER is typically expressed in internal membranes (including the endoplasmic reticulum and Golgi apparatus) under steady state conditions, the mechanism of nuclear PIP3 accumulation remains unclear. Of the two ubiquitous PI3Kinase p110 catalytic subunits, whereas p110β has been associated with multiple nuclear functions [31], we demonstrated that p110α, which is typically associated with cytosolic/plasma membrane signaling [30], was responsible for the estrogen-stimulated translocation of FOXO3. In addition, we observed that inhibition of p110β in the absence of cell stimulation led to FOXO3 translocation that was prevented by p110α inhibition, suggesting a complex interaction between p110α and p110β signaling pathways. Furthermore, whether GPER mediates activation of p110α/PI3Kinase resident in the nucleus or induces its translocation to the nucleus upon activation remains unclear. Madeo et al. have reported that GPER stimulation results in the formation of a complex involving GPER and the EGFR, which together are recruited to the promoter of genes such as cyclin D1 [67]. Although it is not clear how multiple integral membrane proteins are recruited to and localize to DNA promoter elements, this observation suggests a mechanism by which estrogen-mediated GPER stimulation could potentially result in EGFR-dependent transactivation of PI3Kinase in the nucleus.

Mutliple studies have reported that GPER represents a novel mechanism by which tamoxifen resistance can arise in ERα-dependent breast cancer cells [51–54]. Our results provide a possible mechanism for this resistance, as tamoxifen, ICI182,780 and raloxifene each stimulated FOXO3-GFP translocation to the cytoplasm in a GPER-dependent manner. Thus, although SERMs and SERDs clearly inhibit the proliferation of MCF7 cells, the stimulatory actions of these therapeutic agents on GPER, resulting in the inactivation of FOXO3a and its proapoptotic signaling, may provide the constitutive prosurvival signals, that in time with other alterations and mutations in signaling pathways results in resistance to ERα-targeted therapies. This effect of GPER on survival signaling was demonstrated as G-1, similar to estrogen, greatly inhibited the activation of caspases (under serum/estrogen-deprived conditions that result in the induction of apoptosis) and is supported by results demonstrating that knockdown of FOXO3a in MCF7 cells results in enhanced anchorage-independent growth [64].

## Conclusions

In conclusion, we have revealed a novel function of GPER activation, namely the inactivation of FOXO3a, a consequence of which may be the enhanced survival of breast cancer cells being targeted with anti-hormone therapies (SERMs and SERDs), which through their activation of GPER results in FOXO3a downregulation and inhibition of proapoptotic signaling, in opposition to their intended and primary functions in targeting ERα. Although the interactions of ERα and FOXO3a are clearly complex and interdependent, our results suggest that inhibiting GPER activity during the course of SERM/SERD treatment could represent a novel mechanism to reduce the occurance of resistance to these drugs. Alternatively, the development of SERMs/SERDs lacking cross-reactive agonism towards GPER could represent the next generation of anti-hormone therapy.

## Abbreviations

EGFR: Epidermal growth factor receptor
ER: Estrogen receptor
FOXO3: Murine Forkhead box O3
FOXO3a: Human Forkhead box O3a
GPER: G protein-coupled estrogen receptor
MAPK: Mitogen-activated protein kinase
PI3Kinase: Phosphoinositide 3-kinase
PIP3: Phosphatidylinositol (3,4,5)-trisphosphate
SERD: Selective estrogen receptor downregulator
SERM: Selective estrogen receptor modulator
